# Atypical Frontotemporal Dementia Associated With *SQSTM1* Gene Mutation: A Clinicopathological Case

**DOI:** 10.1111/neup.70029

**Published:** 2025-10-12

**Authors:** Christian Espinoza‐Vinces, María Victoria Zelaya Huerta, Valle Coca Pueyo, Genoveva Montoya‐Murillo, Ana Patiño‐García, Rafael Villino‐Rodríguez, Ainhoa Atorrasagasti‐Villar, Javier Arbizu, Mario Riverol

**Affiliations:** ^1^ Department of Neurology Clínica Universidad de Navarra Pamplona Spain; ^2^ Department of Pathology University Hospital of Navarra Pamplona Spain; ^3^ Brain Bank of Navarra Navarrabiomed, Fundación Miguel Servet Pamplona Spain; ^4^ Navarra Institute for Heath Research (IdiSNA) Pamplona Spain; ^5^ Department of Pediatrics and Clinical Genetics Clínica Universidad de Navarra Pamplona Spain; ^6^ Department of Nuclear Medicine Clínica Universidad de Navarra Pamplona Spain

**Keywords:** dementia, FTLD, *SQSTM1*, TDP‐43

## Abstract

A 78‐year‐old man presented with a six‐year history of progressive memory decline, initially manifesting as recent memory impairment and mild anomia, which gradually evolved into motor clumsiness, gait disturbances, language difficulties, behavioral changes, and late‐onset parkinsonism. He had been diagnosed with Paget disease of bone (PDB) at the age of 45. Brain MRI revealed asymmetric left anterior temporal atrophy, while [^18^F]‐Fluorodeoxyglucose (FDG) PET demonstrated frontotemporal hypometabolism, predominantly on the left side, with marked involvement of both temporal poles and greater hypometabolism in the left temporal pole. A negative amyloid PET scan supported a diagnosis of frontotemporal dementia (FTD). Genetic analysis identified an *SQSTM1* gene mutation (c.1210A>G; p.(Met404Val)). Post‐mortem examination confirmed frontotemporal lobar degeneration with atypical TDP‐43 protein distribution, alongside tau and Lewy body pathology. This case exemplifies an atypical presentation of FTD, characterized by amnestic onset with subsequent language and behavioral involvement, thereby broadening the recognized clinical spectrum of *SQSTM1*‐associated FTD. The coexistence of parkinsonism and PDB, alongside mixed proteinopathies, underscores the phenotypic heterogeneity of *SQSTM1* mutations. These findings emphasize the importance of considering prominent memory impairment, semantic deficits, and parkinsonism as potential manifestations in this genetic form and highlight the need for comprehensive clinical, genetic, and neuropathological evaluation to improve diagnosis and inform therapeutic strategies.

**Abbreviations**
ACMG/AMP: American College of Medical Genetics and Genomics/association for molecular pathology
ALS: amyotrophic lateral sclerosis
AT8: antibody specific for phosphorylated tau
bvFTD: behavioral variant frontotemporal dementia
*CHCHD10*
: coiled‐coil‐helix‐coiled‐coil‐helix domain containing 10
*CHMP2B*
: charged multivesicular body protein 2B
CYLD: cylindromatosis
*DCTN1*
: dynactin subunit 1
DNs: dystrophic neurites
FDG‐PET: fluorodeoxyglucose positron emission tomography
FLAIR: fluid‐attenuated inversion recovery
FTD: frontotemporal dementia
FTD/ALS: frontotemporal dementia/amyotrophic lateral sclerosis
FTLD: frontotemporal lobar degeneration
FTLD‐TDP: frontotemporal lobar degeneration with TDP‐43 pathology
*FUS*
: fused in sarcoma
*GRN*
: granulin
HE: hematoxylin–eosin staining
*HNRNPA2B1*
: heterogeneous nuclear ribonucleoprotein A2/B1
LC3: microtubule‐associated proteins 1A/1B light chain 3
Lewy bodies: proteinaceous inclusions associated with parkinsonism
*MAPT*
: microtubule‐associated protein tau
MbL: Medical & Biological Laboratories Co.
MMSE: mini‐mental state examination
MND/ALS: motor neuron disease/amyotrophic lateral sclerosis
MRI: magnetic resonance imaging
NCIs: neuronal cytoplasmic inclusions
NF‐kappa: nuclear factor kappa‐light‐chain‐enhancer of activated B Cells
NGS: next‐generation sequencing
NII: neuronal nuclear inclusions
p62: sequestosome 1 protein
PDB: paget's disease of bone
PET: positron emission tomography
PM: pathogenic moderate (ACMG classification)
PP: pathogenic supporting (ACMG classification)
PS: pathogenic strong (ACMG classification)
*PSEN1*
: presenilin 1
*PSEN2*
: presenilin 2
pTDP‐43: phosphorylated TAR DNA‐binding protein 43
*SQSTM1*
: sequestosome 1
svPPA: semantic variant primary progressive aphasia
*TARDBP*
: TAR DNA‐binding protein
*TBK1*
: TANK‐binding kinase 1
TDP‐43: TAR DNA‐binding protein 43
TIA1: T‐cell intracellular antigen 1
*TUBA4A*
: tubulin alpha 4A
*UBQLN2*
: ubiquilin 2
*VCP*
: valosin‐containing protein

## 1 Introduction

Frontotemporal dementia (FTD) is the second most common dementia subtype in individuals under 65 years old, characterized by selective frontal and temporal lobe atrophy [1]. The term frontotemporal lobar degeneration (FTLD) with or without motor neuron disease/amyotrophic lateral sclerosis (MND/ALS) unifies a range of clinically diverse disorders.

FTD is a genetically and clinically heterogeneous neurodegenerative disorder. The most common genetic mutations associated with FTD include *MAPT*, *GRN*, and *C9orf72* [2]. In addition, other genes such as *VCP*, *CHMP2B*, and *TBK1* have been implicated in its pathogenesis, with *TBK1* accounting for approximately 1% of the total FTD/ALS genetic load [3]. Although less frequent, mutations in genes like *CHMP2B* and *CHCHD10* are also relevant and should be considered in comprehensive genetic evaluations [4].

Although less frequent, mutations in the *sequestosome 1* (*SQSTM1*) gene have also been identified, further emphasizing the genetic variability of this condition [5].

The *SQSTM1* gene, located on chromosome 5, encodes p62, a protein involved in autophagy, protein degradation, and NF‐kappa activation [6]. p62 binds polyubiquitinated proteins for proteasomal degradation and aids autophagy through its LC3‐interacting region [7]. Initially linked to Paget's disease of bone (PDB), *SQSTM1* mutations are also associated with ALS, FTD, and FTD‐ALS, involving p62 and TDP‐43 inclusions [5, 8].

In the molecular pathological classification of FTLD, abnormal intracellular protein deposits, including hyperphosphorylated tau, TDP‐43, and FUS proteins, are key in identifying subtypes. Notably, TDP‐43 accumulation is a defining characteristic in a significant subset of FTLD cases, providing crucial markers for disease classification [5].

Despite advances in using molecular markers like ubiquitin/p62 immunostaining, the neuropathological classification of FTLD remains complex, especially in postmortem analyses [9]. Diagnostic challenges arise when clinical presentations do not align with pathological findings, underscoring the need for refined methods to better correlate clinical and pathological phenotypes in FTLD diagnosis.

## 2 Clinical Summary

A 78‐year‐old right‐handed man consulted our department due to a six‐year history of progressive memory impairment and slight difficulty recalling object names, followed by language difficulties that gradually impacted his daily activities and instrumental tasks. He had completed 15 years of formal education, achieving a technical qualification, and had a medical history of PDB, diagnosed at the age of 45, as well as dyslipidaemia. Born to non‐consanguineous parents, he has a paternal uncle with an age‐related language disorder. According to the family, language difficulties, characterized by impaired comprehension, appeared shortly after the initial memory problems, accompanied by motor clumsiness, gait impairment, and mood changes, including irritability, apathy, and mild disinhibition.

The initial neurological examination revealed inattention and perseveration, with preserved praxis, normal muscle strength, and no sensory deficits. Six years later, the patient exhibited mild generalized bradykinesia and gait impairment, characterized by reduced arm swing, whereas the rest of the neurological examination remained normal.

At baseline, the neuropsychological assessment showed mild amnestic cognitive impairment, mainly affecting verbal and visual episodic memory, with a Mini‐Mental State Examination (MMSE) score of 26 out of 30. At this stage, language was relatively preserved, with appropriate fluency, preserved comprehension even for complex commands, intact repetition, and only subtle difficulties in naming low‐frequency objects. Over time, the patient developed a decline involving multiple cognitive domains, including episodic and marked semantic memory. Language impairment became more evident, with prominent naming difficulties, phonemic paraphasias, and reduced understanding of single words, which further limited daily activities, whereas fluency and repetition remained relatively preserved. Language abilities were assessed using recognized tools, including the Boston Naming Test, semantic verbal fluency (animals), and phonemic verbal fluency (letter “P”). During this evaluation, speech was noted to be paraphasic, with limited informational content, and a severe impairment of semantic knowledge was observed, as the patient showed difficulties in understanding and categorizing common objects. Behavioral symptoms also emerged, most notably subtle disinhibition, such as occasional use of inappropriate words in social settings and mildly intrusive behaviors toward family members, for example, interrupting conversations or disregarding personal space. At follow‐up, the MMSE score had declined to 22 out of 30. Taken together, these clinical features support a diagnosis of FTD, with progressive language and memory impairment, prominent semantic deficits, and behavioral changes while not aligning with any established FTD subtype.

**FIGURE 1:**
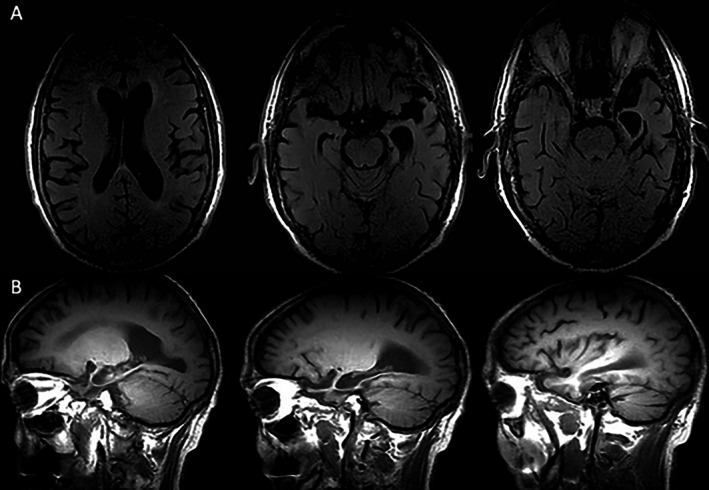
Brain MRI showing atrophy of the left temporal lobe, including both medial structures with amygdala‐hippocampal involvement and anterior regions. (A) Fluid‐attenuated inversion recovery (FLAIR) sequence, axial plane. (B) T1‐weighted MRI scan, sagittal plane.

**FIGURE 2:**
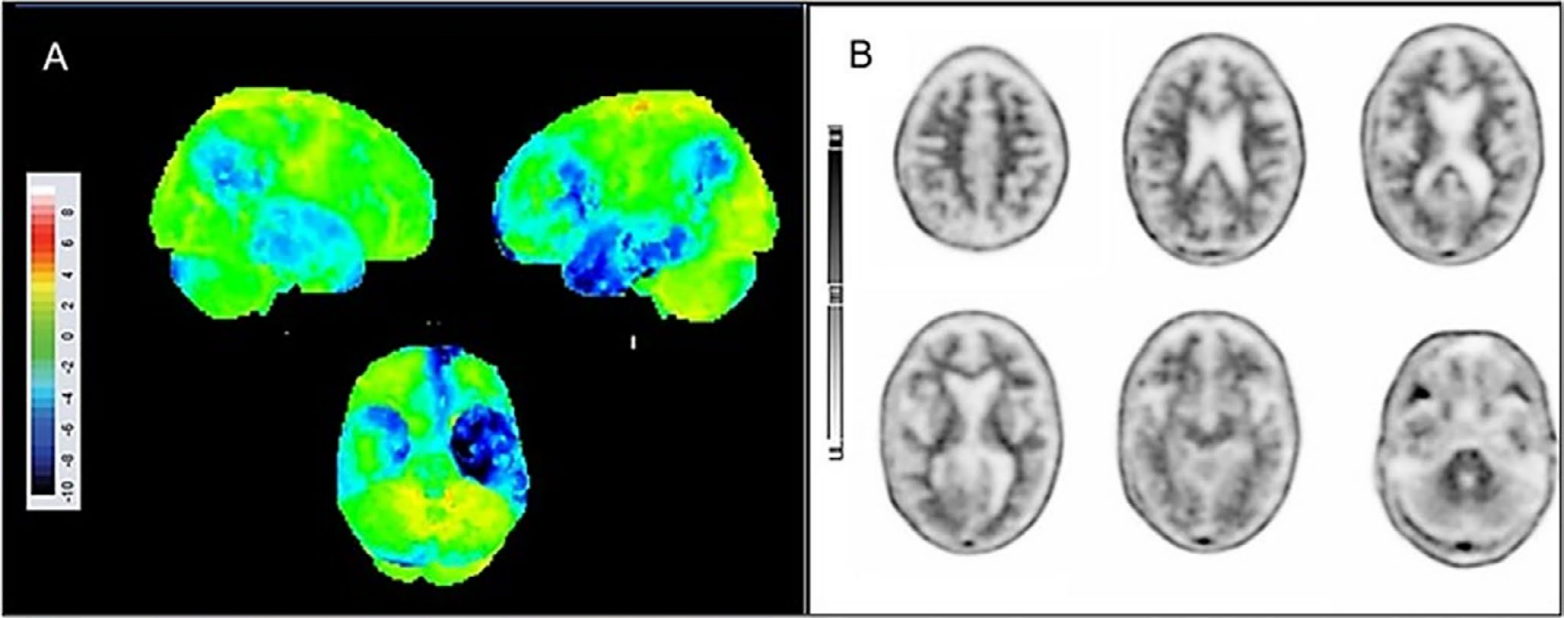
[^18^F]‐FDG PET and amyloid PET images. (A) Statistical surface projections of [^18^F]‐FDG‐PET after database comparison with a normal population adjusted by age. Significant cortical hypometabolism (> 2 SD in blue) is observed in both temporal poles, with a predominance in the left temporal pole, as well as in the left frontal and fronto‐insular regions. (B) [^18^F]‐Florbetapir amyloid PET scan was negative for elevated β‐amyloid cortical deposition (−30.7 Centiloids).

Routine blood tests for dementia, including vitamins, were normal. Brain magnetic resonance imaging (MRI) showed atrophy in the anterior and medial left temporal lobe, including the amygdala and hippocampus (Figure 1A,B). The [^18^F]‐Fluorodeoxyglucose (FDG) brain positron emission tomography (PET) scan exhibited frontotemporal hypometabolism predominantly on the left side, affecting both temporal poles with greater involvement of the left temporal pole (Figure 2A), whereas the brain amyloid PET scan was negative (Figure 2B). The patient died at 85 after progressive deterioration.

To better characterize the clinical phenotype, genetic analysis was performed using next‐generation sequencing (NGS) targeting genes associated with frontotemporal dementia (FTD), including *CCNF*, *CHMP2B*, *CYLD*, *DCTN1*, *MAPT*, *GRN*, *HNRNPA2B1*, *PLA2G6*, *SQSTM1*, *TARDBP*, *TBK1*, *TIA1*, *TUBA4A*, *PSEN1*, *PSEN2*, *UBQLN2*, *CHCHD10*, and *VCP*. The study identified a heterozygous c.1210A>G (p.Met404Val) variant in the *SQSTM1* gene (NM_003900.5), classified as pathogenic. This classification was based on the ACMG/AMP criteria PS3, PM2, PM5, PP3, and PP5, integrating functional data, population frequency, in silico prediction, and previous variant reports. Given the autosomal dominant inheritance pattern of *SQSTM1* variants, this heterozygous mutation was considered diagnostic.

## 3 Pathological Findings

### 3.1 Materials and Methods for Autopsy

According to Spanish Law 14/2007 on Biomedical Research, an informed written consent form from the Neurological Tissue Bank of the Navarra Health Service was obtained for diagnostic and research purposes. Immediately after removal from the skull, the right cerebral hemisphere was progressively frozen, sliced into 1–1.5 cm‐thick coronal sections, and stored at −80°C. The left cerebral hemisphere was placed in 10% formalin for 4 weeks, and representative brain areas were selected as previously described [10]. After macroscopic examination, immunohistochemistry analysis was performed in different brain regions using specific antibodies against Tau protein, β‐amyloid, pTDP‐43, PrP, α‐synuclein, and P62. Four micrometer thick sections were stained with hematoxylin and eosin and processed for immunohistochemistry.

### 3.2 Immunohistochemistry

Formalin‐fixed, 4‐μm‐thick sections were mounted on slides and de‐waxed. After conducting a routine antigen retrieval protocol, sections were incubated for 1 h with the following primary antibodies: Aβ‐amyloid (1/200, Dako, Glostrup, Denmark), anti‐AT8 (1/100, Immunogenetics, Ghent, Belgium), anti‐α‐synuclein (1/50, NCL‐L‐ASYN; Leica Biosystems, Wetzlar, Germany), anti‐TDP‐43 (1/8000, Abcam, Cambridge, United Kingdom), Ubiquitin (1/700, FPM1; Leica Biosystems) and anti‐P62 (1/100, *SQSTM1*, MBL, Japan). The reaction product was visualized using an automated slide immunostainer (Leica Bond Max) with Bond Polymer Refine Detection (Leica Biosystems Newcastle Ltd) and counterstaining with hematoxylin–eosin.

### 3.3 Autopsy Findings

**FIGURE 3:**
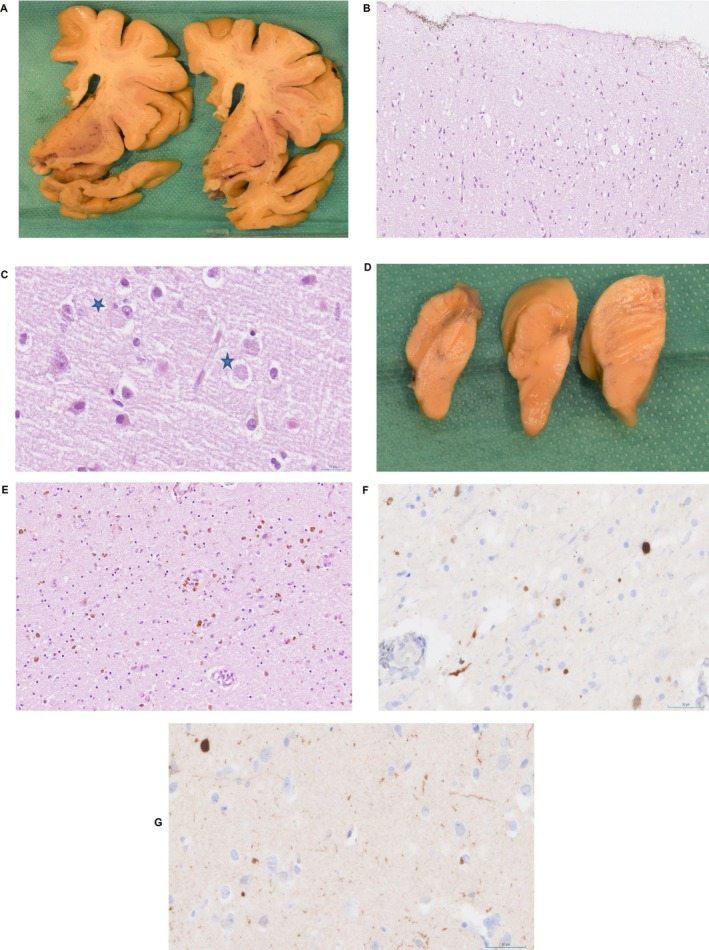
(A) Macroscopic findings showing mild frontotemporal cortical atrophy with predominant involvement of the amygdala. (B) Cortical superficial spongiosis (motor cortex 20×). (C) Pale cortical Lewy bodies (indicated by blue star in cingulate cortex 40×). (D) Macroscopic findings showing severe depigmentation of substantia nigra and locus coeruleus. (E) Severe loss of neurons and signs of neuronophagy in midbrain (HE 20×). (F–G) Alpha‐synuclein immunohistochemistry. (F) At low magnification, presence of Lewy bodies and scattered neurites in substantia nigra (40×). (G) Few Lewy bodies and neurites present in cingulate cortex (40×).

The post‐mortem neuropathology study showed mild frontal and temporal cortical atrophy, with a clear predominance of amygdalar and limbic involvement, as well as marked ventricular dilatation (Figure 3A). In the microscopy study of the motor cortex, we observed superficial microvacuolation of neuropil (Figure 3B). This histological change was striking in the cingulate cortex, where scattered α‐synuclein–positive inclusions compatible with pale Lewy bodies were identified, although they were relatively few in number (Figure 3C). Mild to moderate signs of gliosis and neuronal loss were observed in the frontal and temporal cortical areas as well. The substantia nigra and locus coeruleus exhibited severe depigmentation (Figure 3D).

The study of the brainstem demonstrated a decreased number of dopaminergic neurons with signs of neuronophagy in the substantia nigra (Figure 3E) and intraneuronal inclusions in the motor dorsal nucleus at the level of the medulla oblongata. Severe astrogliosis and loss of neurons were observed in the amygdala and hippocampus. Ischemic lesions, such as lacunar infarcts, were identified in the centrum semiovale of white matter. The basal ganglia showed hemosiderin pigment in the putamen, astrogliosis in the thalamus with intranuclear inclusions, and small bleeds in the pallidum.

Alpha‐synuclein immunohistochemistry revealed Lewy bodies and scattered neurites in the substantia nigra (Figure 3F), with fewer neuronal inclusions in the cingulate cortex (Figure 3G). No evidence of immunoreaction was present in other anatomical regions. Tau immunopositivity was present in thorn‐shaped astrocytes in the white matter of periventricular areas, with mild neurofibrillary degeneration (Braak stage I) consistent with aging‐related tau astrogliopathy (ARTAG) [11].

Beta‐amyloid immunostaining showed no pathological deposits. Immunohistochemistry for pTDP‐43 demonstrated the predominant protein deposit in the brain. Staining for ubiquitin and p62 was also positive, as expected across FTLD‐TDP subtypes, though it did not aid in subtype differentiation.

**FIGURE 4:**
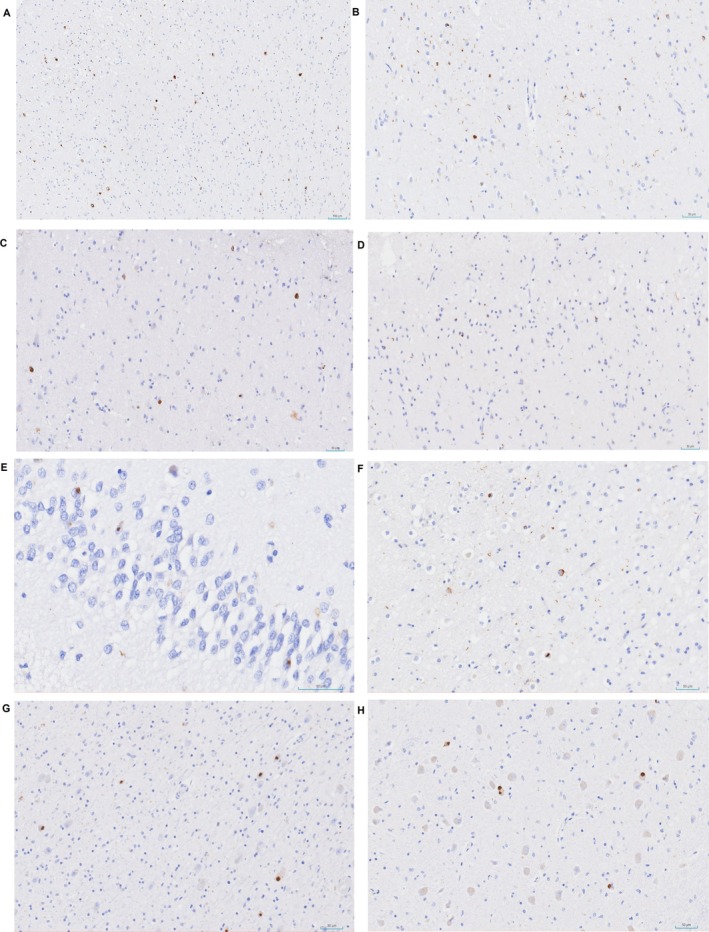
Microscopic findings of immunostaining for p‐TDP43. (A) Positive inclusions in superior and inferior cortical layers (cingulate cortex 10×). (B) Frequent short dystrophic neurites and NCI (superior cortical layer in cingulate cortex 20×). (C) Neuronal cytoplasmic inclusions (NCIs) with round morphology (motor cortex 40×). (D) NCIs with circumferential morphology and short neurites (medial frontal lobe 20×). (E) NII in fascia dentata of hippocampus (40×). (F) NII with circumferential, cat eye morphology and short neurites in temporal cortex (20×). (G) NII with round morphology in striatum (20×). (H) Brainstem (olivary nucleus of medulla oblongata) (20×).

The most frequent immunomorphology for pTDP43 was neuronal cytoplasmic inclusions (NCIs) with circumferential morphology in all the cortical layers (Figure 4A,B) of the frontal lobe; we also observed that short dystrophic neurites were evident in the superior cortical layers of the cingulate cortex (Figure 4B). Neuropil deposits, found as thin and short threads, were also present in the frontal, temporal, and limbic areas, distributed in superficial and deep cortical layers, with extension to the anterior horn of the spinal cord. Few oligodendroglial deposits were seen, reminiscent of coiled bodies.

Some immunopositivity caught our attention in the cingulate cortex, precentral region, thalamus, and olivary nucleus of the brainstem; their morphology appeared as compact globular inclusions in neurons (Figure 4C,D). Occasional neuronal nuclear inclusions (NII) were also noted. NII were found in the fascia dentata of the hippocampus (Figure 4E), and in the temporal cortex, we spotted a cat‐eye morphology inclusion (Figure 4F). In the striatum and olivary nucleus of the medulla oblongata, inclusions with round morphology were observed (Figure 4G,H).

Histopathological findings were consistent with FTLD‐TDP, without fully aligning with the harmonized TDP‐43 classification system [12]. The presence of deposits in NII suggested a possible FTLD‐TDP type D, though the absence of lentiform NII was atypical.

Numerous NCIs and short dystrophic neurites in neocortical regions, particularly within the upper cortical layers, are consistent with FTLD‐TDP type A, displaying some atypical features. NII with round or cat‐eye morphology was observed but is not typical of type A, and no deposits were found in the molecular layer of the hippocampus. Although subcortical, hippocampal, and brainstem regions showed variable NCI morphologies, the neocortical pattern was considered the primary criterion for classification. The variable distribution of inclusions was considered unclassified, without fitting any established morphological subtype.

These findings are illustrated in Figures 3 and 4, showing prominent features resembling type A alongside variable atypical inclusions. Western blot analysis, which could have further refined the molecular categorization of FTLD‐TDP, was not available in our center.

## 4 Discussion

We report a case of late‐onset dementia with an initial amnesic presentation resembling Alzheimer's disease, which was soon followed by prominent language impairment that became the predominant clinical feature. Over time, the patient developed progressive semantic deficits as the leading symptom, accompanied by behavioral changes such as disinhibition. During follow‐up, the patient also exhibited parkinsonism and had a prior history of PDB, further underscoring the atypical phenotype. Genetic testing revealed a pathogenic heterozygous variant c.1210A>G (p.(Met404Val)) in the *SQSTM1* gene, a mutation of low prevalence in FTLD.

In accordance with the genetic heterogeneity of frontotemporal lobar degeneration, our analysis comprehensively covered common and rare genes implicated in FTD and amyotrophic lateral sclerosis, including *TBK1, CHMP2B*, and *CHCHD10*, all of which were negative in this patient, supporting the pathogenic role of the *SQSTM1* variant identified [2].

Mutations in the *SQSTM1* gene impair p62's function in transporting proteins for proteasomal degradation, resulting in cytoplasmic protein accumulation and inclusion body formation, which contribute to tissue degeneration [13]. Notably, p62 co‐localizes with tau, alpha‐synuclein, TDP‐43, and fused‐in‐sarcoma proteins in inclusions linked to various neurodegenerative disorders, thereby connecting SQSTM1 dysfunction with TDP‐43 pathology [14].

This multifunctional interaction highlights p62 as a central element in the pathogenesis of proteinopathies, potentially influencing the expression and distribution of pathological proteins beyond TDP‐43, including coexisting pathologies such as Lewy bodies, as observed in our case [15]. In this context, the clinical variability and the convergence of pathological features found in patients with *SQSTM1* mutations, such as the simultaneous presence of TDP‐43 and alpha synuclein pathology, may reflect the impact of p62 dysfunction on intracellular protein regulation [16].

Our case demonstrated atrophy predominantly in the left medial temporal lobe, with more subtle involvement of the left anterior temporal region, whereas the right temporal pole showed comparatively minor changes, potentially accounting for the observed behavioral features [17]. This highlights a distinct phenotype among patients with SQSTM1 mutations, contrasting with previous reports that primarily described right‐sided atrophy affecting prefrontal, orbital, and insular regions, often with relative preservation of the temporal lobes on brain MRI [18]. Table 1 summarizes the reported *SQSTM1* mutations and their associated clinical phenotypes from the literature, providing a context for comparison with our findings.

**TABLE 1:** Comparison of clinical, imaging, and neuropathological features in reported cases of FTD associated with *SQSTM1* mutations.

References	Number of patients	SQSTM1 mutation	Age at onset	Clinical phenotype	Behavioral symptoms	Motor involvement	Association with bvFTD	Brain imaging	Prognosis	Neuropathological findings
[19]	Multiple families, 4 main mutations in 4 families	p.A33V, p.P387L, p.A381V, p.P392L	48–73 (range across families)	Familial bvFTD and FTD‐ALS (some with Paget's disease)	Disinhibition, rituals, verbal stereotypies, apathy, social avoidance	Some extrapyramidal signs, no ALS in some families, ALS in one proband	Yes, in multiple affected relatives	MRI: cortical and perisylvian atrophy, white matter lesions; SPECT: hypoperfusion of frontal/temporal lobes; DaT scan normal	Variable, some died between ages 70–75 years	Not available
[20]	Multiple (listed mutations)	p.Glu396*, p.Arg212Cys, p.Pro387Leu, p.Pro232Thr, and others	41–73	FTLD spectrum: bvFTD, FTLD‐ALS, PNFA, PSP	Behavioral variant FTD symptoms in many	Motor neuron disease in some, PSP in one	Yes	Various MRI/SPECT findings reported	Variable	TDP‐43 pathology with phospho‐TDP‐43 inclusions; distinct pathology for p.Glu396* and p.Arg212Cys; p62 immunoreactivity reduced or absent in some brain regions
[21]	1	S224X	59	Atypical FTD with memory difficulties and personality changes	Forgetfulness, apathy, sluggishness, reticence	Slow gait, occasional falls	Not formally bvFTD, but overlapping symptoms	Mild frontal and temporal atrophy; no progression after 10 months	MMSE declined from 20 → 12 in 10 months; progressive cognitive decline	Suggested SQSTM1 haploinsufficiency and absent p62 protein
[5]	2	P1: p.Glu396* P2: p.Arg212Cys + C9orf72 expansion	Patient 1: 43 Patient 2: 63	Patient 1: bvFTD Patient 2: bvFTD with parkinsonism and bulbar symptoms	Patient 1: Apathy, disinhibition, hyperorality, paranoia Patient 2: Disinhibition, mistrust, impulse loss, paranoid delusions	Patient 1: No parkinsonism or ALS Patient 2: Parkinsonism (DAT+), bulbar, corticospinal signs	Yes (both)	Patient 1: Frontal diffuse atrophy; elevated tau, normal amyloid Patient 2: Generalized frontal atrophy, enlarged ventricles; PET frontal hypometabolism	P1: 32 months (death from aspiration) P2: 2.5 years (death from MI)	P1: TDP‐43 type B with oligodendroglial inclusions P2: TDP‐43 type A, p62 pathology, C9orf72 poly(GP) inclusions
[17]	10	p.L238del (2), p.P392L, p.P387L, p.A33, p.E319K, p.P348L, p.P439L, p.T430P, p.D329G	Mean 66 (44–79)	bvFTD (7), PNFA (3), 1 FTD + Paget	Psychotic symptoms in 1	1 ALS, 1 Paget's, 2 parkinsonism	Yes (7/10 bvFTD)	Asymmetric right fronto‐orbital and insula GM loss; premotor/motor areas involved	Mean duration 4 ± 2.4 years	Not available
[22]	1	p.392L	54	Episodic memory impairment (Alzheimer‐like)	Not specified	None reported	No (initially suspected AD)	Cortical atrophy and hypometabolism temporal lobes, no amyloid biomarkers	Slowly progressive; duration not specified	Not available
[23]	1	c.436_462dup p.(Pro146_Cys154dup)	70	Probable bvFTD	Personality disorder since youth, cognitive decline, behavioral disturbances	Not reported	Yes	Not specified	Not specified	Not available

*Note:* The asterisk (*) indicates a missense mutation.

Abbreviations: ALS, amyotrophic lateral sclerosis; bvFTD, behavioral variant frontotemporal dementia; DaT scan, dopamine transporter scan; FTD, frontotemporal dementia; FTLD, frontotemporal lobar degeneration; GM, gray matter; MI, myocardial infarction; MMSE, mini‐mental state examination; MRI, magnetic resonance imaging; P1, patient 1; P2, patient 2; PET, positron emission tomography; PNFA, progressive nonfluent aphasia; PSP, progressive supranuclear palsy; SPECT, single‐photon emission computed tomography.

Clinically, our case aligns with an atypical variant of FTD, with an initial amnesic onset that was quickly superseded by prominent language dysfunction, accompanied by TDP‐43 pathology, consistent with previous studies [5]. Although the initial amnestic phase briefly resembled an Alzheimer‐like profile, semantic deficits rapidly became predominant, and several years later behavioral disturbances, particularly disinhibition, emerged. This pattern illustrates the clinical complexity and heterogeneity of FTD without assigning the patient to a predefined subtype.

Importantly, the early amnestic presentation, consistent with prior reports of FTD linked to *SQSTM1* variants, reinforces the relevance of memory deficits as part of the clinical spectrum [22]. Beyond confirming this early phenotype, our case provides detailed longitudinal documentation of its evolution into a mixed semantic and behavioral profile with bilateral temporal involvement. These observations underscore the broad heterogeneity of pathogenic *SQSTM1* alterations and illustrate that atypical presentations can be meaningfully captured through careful, phenotype‐focused description that complements existing subtype frameworks. Careful longitudinal phenotypic characterization of such atypical cases, combined with neuropathological confirmation, is essential to establish reference cohorts for future research and to delineate the full clinical spectrum associated with changes in the *SQSTM1* gene.

Furthermore, the patient was initially diagnosed with PDB at age 45, which represents the first clinical association reported with *SQSTM1* gene mutations [13]. PDB often precedes the onset of FTD, highlighting the importance of considering *SQSTM1* mutations in relevant clinical scenarios [24]. Although mutations in the VCP gene also cause a multisystem proteinopathy that includes inclusion body myopathy, PDB, and FTD [25], PDB is more consistently observed in patients with *SQSTM1* mutations than in those with VCP mutations, despite up to 51% of VCP mutation carriers developing PDB [26].

Previous studies have reported that the average time from diagnosis to death for patients with the *SQSTM1* genotype is 7.2 ± 4.8 years, which is shorter compared to those with *GRN* and *MAPT* mutations [27]. Our case aligns with this prognosis but further illustrates the phenotypic variability and clinical progression that may be expected.

Parkinsonism is not commonly linked to *SQSTM1* mutations; however, our patient exhibited significant neuronal loss in the substantia nigra compacta and reticulata [5]. Although TDP‐43 pathology may be more widespread than clinical symptoms suggest, the presence of scattered α‐synuclein–positive inclusions at the limbic stage, most likely related to the patient's advanced age, was also identified [5]. We acknowledge that the amount of α‐synuclein pathology was limited, and therefore the severe nigral depigmentation cannot be solely explained by Lewy body disease. Based on the overall pathological findings, we attribute the parkinsonian signs primarily to striatonigral denervation associated with the combined effect of α‐synuclein and TDP‐43 pathology, the latter of which is increasingly recognized as a contributor to nigral degeneration and parkinsonism in FTLD [28].

In conclusion, we describe an atypical variant of FTD associated with a *SQSTM1* mutation, characterized by an initial amnestic onset followed by predominant language impairment and subsequent behavioral changes, in the context of parkinsonism and PDB. These findings broaden the recognized clinical and genetic spectrum of FTD‐PDB and underscore the importance of capturing heterogeneous and atypical phenotypes, including those with early memory deficits evolving into combined semantic and behavioral profiles.

## Ethics Statement

This study was conducted in accordance with the ethical principles set forth in the Declaration of Helsinki (1964) and its subsequent amendments. In compliance with Spanish Law 14/2007 on Biomedical Research, written informed consent was obtained through the Neurological Tissue Bank of the Navarra Health Service for both diagnostic and research purposes.

## Consent

The patient's family provided informed consent for the publication of this case report, as well as written authorization for autopsy and postmortem analysis for research use.

## Conflicts of Interest

The authors declare no conflicts of interest.

## Data Availability

The data that support the findings of this study are available from the corresponding author upon reasonable request.
